# Pathfinder studies: a novel tool for process mapping data-driven health research to build global research capacity

**DOI:** 10.1186/s12874-025-02638-7

**Published:** 2025-08-07

**Authors:** Aashna Uppal, Frank Kagoro, Luciana Monteiro-Krebs, Flavia Thedim Costa Bueno, Larissa Pruner Marques, Sofonias Kifle Tessema, John Amuasi, Matthew Retford, Bonny Baker, Mercedes Rumi, Aliya Naheed, Elvis Temfack, Cristiani Vieira Machado, Agklinta Kiosia, Paul Kingpriest, Trudie Lang

**Affiliations:** 1 The Global Health Network, Centre for Tropical Medicine and Global Health, Nuffield Department of Medicine, University of Oxford, Oxford, UK; 2 Afya Research and Data Consultancy (Afredac), Dar es Salaam, Tanzania; 3 University of Cape Town, Cape Town, South Africa; 4 Oswaldo Cruz Foundation (Fiocruz), Rio de Janeiro, Brazil; 5 Federal University of Rio Grande do Sul, Porto Alegre, Brazil; 6 Africa Centres for Disease Control and Prevention (Africa CDC), Addis Ababa, Ethiopia; 7 Kumasi Center for Collaborative Research in Tropical Medicine, Kwame Nkrumah University of Science and Technology (KNUST), Kumasi, Ghana; 8 Bernhard Nocht Institute of Tropical Medicine (BNITM), Hamburg, Germany; 9 Health Data Research Global (HDR Global), London, UK; 10 International Centre for Diarrhoeal Disease Research, Bangladesh (icddr,b), Dhaka, Bangladesh; 11 Department of Metabolism, Digestion and Reproduction, Division of Digestive Diseases, Section of Nutrition, Faculty of Medicine, Imperial College, London, UK

**Keywords:** Public health research methods, Data science, Research inequality

## Abstract

**Background:**

There is vast global inequality regarding where health research happens, who leads the research, and who benefits from the evidence. Globally, wealthier nations drive and influence data-driven research and how it is structured institutionally. Key barriers to high-quality research being undertaken in and led by low-resource settings are well reported. These barriers persist, thereby perpetuating a lack of locally generated data and/or evidence to tackle diseases that bring the greatest burden. Our aim was to design a tool to capture best practices in the production of data-driven health research, to advance both quality and quantity of research being conducted where it is needed most.

**Methods:**

An expert group of senior global health researchers from Asia, Africa, Europe, and Latin America and the Caribbean (LAC) convened to discuss potential solutions to addressing this imbalance in both quality and quantity of global health research. This study documents how a novel approach was developed, informed by this discussion, to support research teams in low-resource settings. The new approach, called “Pathfinder”, is a process-mapping tool wherein teams document key steps of their research projects flow to produce quality data and subsequent studies.

**Results:**

The Pathfinder methodology is a novel tool to be used alongside planned studies to guide teams through each step of their research, from setting their research question, to identifying the best methods needed to complete each step, to translating research outputs into impactful policy and practice. It is a standardized framework, which can be applied or adapted to specific settings for research teams track to key steps, challenges, solutions, and tools throughout their planned study’s process. Pathfinders can also be applied to studies that have already been completed, retroactively documenting their key components. Several global research institutes are piloting the Pathfinder methodology.

**Conclusions:**

Pathfinders can help inform future studies by capturing best practices, thereby removing barriers to research, and addressing global inequality in this domain. Specifically, Pathfinders can help identify the methods and skills needed for teams to produce safe, ethical, and accurate data-driven health research.

**Supplementary Information:**

The online version contains supplementary material available at 10.1186/s12874-025-02638-7.

## Background

Determining effective disease prevention, management, and treatment requires a connected ecosystem of health data to be collected accurately and ethically through different forms of health research [1]. Generating this vital evidence requires improved uptake of data-driven health research across the globe and in every care setting [2]. However, there is great inequality in where research studies happen and where data is captured, used, and shared; this results in further inequality in who leads the studies and who benefits from the evidence generated [3, 4].

There is broad recognition that data collection and analysis for the purpose of health research is often not undertaken in many settings, or by some groups of healthcare professionals, because research and associated data analysis methods are deemed to be too cumbersome or inaccessible under specific resource constraints [3, 4]. For example, clinical trial methodology – coined as the gold standard in health research – is resource intensive; training staff in running such studies and analysing associated data also requires sufficient resources and funds. In addition to inaccessible methods and training, inaccessible infrastructure for navigating regulatory procedures may further inhibit researchers from undertaking prospective research or asking new questions of existing data [3–6]. That is not to say that there is a lack of robust health data in these settings; in fact, there are vast amounts of health data, which are often not being analysed to ask questions that can guide disease management, prevention and treatment [1, 2, 7].

A study involving over 7,000 researchers across low- and middle-income countries showed that there was a high degree of overlap in where difficulties arose, either directly in undertaking primary health research, or indirectly in the environment that supported the health research process [3]. Common areas of difficulty were related to data: including challenges in data collection and capture, data standardization, and the technical aspects of infrastructure related to data management, analysis and sharing [3]. Indirect elements such as data privacy, governance, trust, and engagement with the community were identified as potential amplifiers of gaps in data use and reuse in health research [3].

However, while there are accessible solutions, tools, and training available to address these common challenges, research teams in resource-limited settings often remain unaware and alone in designing, operating, and reporting their studies [4]. Current health research capacity building initiatives focus on leveraging institutional partnerships, particularly those between high-income settings and low- and middle-income settings [8]. Initiatives also focus on participatory research and training of local health workers to ensure research sustainability [9], alongside programs that develop research infrastructure and networking/knowledge sharing opportunities [8].

To complement the impactful initiatives that already exist to advance health research in low-resource settings, we propose that there is merit in documenting common challenges, solutions, and tools encountered by health researchers, across a variety of settings to share best practices and facilitate data-driven health research where it is needed most. Accordingly, we sought to develop a tool to aid research teams – particularly in resource-limited settings – in pursuing locally led data-driven health research by documenting these key components in the production of data-driven health research. This paper aims to describe the development, methods, and potential applications of this tool.

## Methods

A group of twelve senior global health researchers (FK, FTCB, LPM, SKT, JA, MR, BB, MR, AN, ET, CVM, and TL), each with at least ten years of experience in global health research, convened from major health research institutions in Asia (*n* = 1 institution), Africa (*n* = 2), Europe (*n* = 2), and Latin America and the Caribbean (LAC) (*n* = 1) in January 2023. Expert inclusion was based on leadership from institutions affiliated with The Global Health Network [10], which has over one million members, and has global representation of researchers from resource-limited settings. Further, expert selection was based on a convenience sample, with representation from all regions in which The Global Health Network operates. There was a variety of methodological expertise within this group of researchers, including clinical trial expertise, as well as qualitative and quantitative methodological expertise in observational health research. While all researchers had experience leading studies in resource-limited contexts, we ensured some degree of regional diversity to represent cultural differences in conducting research. Of note, there were no specific incentives provided for participation in this group.

**Fig. 1:**
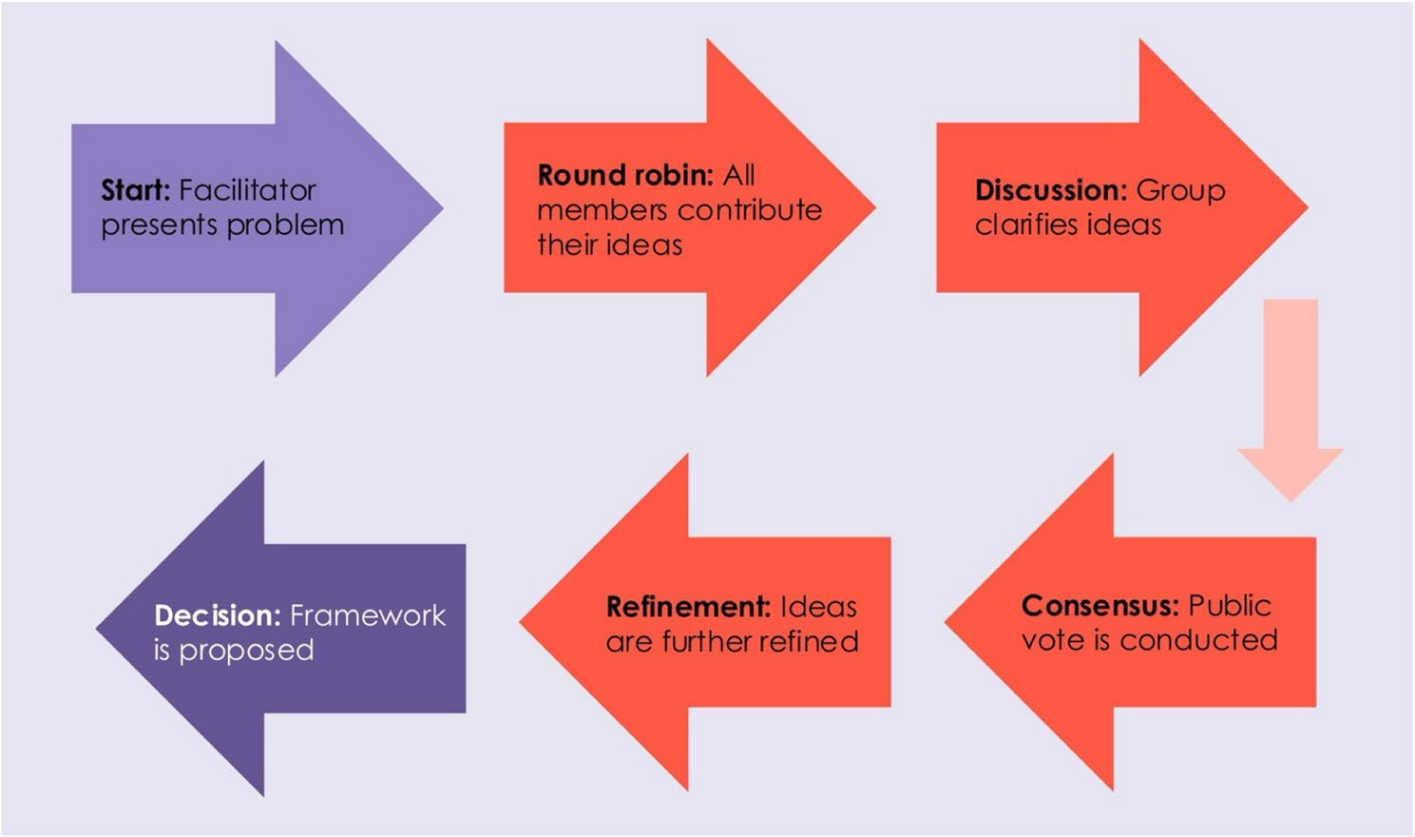
Nominal group technique for consensus building

Accordingly, experts reviewed current evidence on the “study within a trial” concept [8], which is a concept that involves conducting a methodological study embedded within a clinical trial to improve processes of the trial itself; experts then engaged in a single ninety-minute group discussion. This discussion was conducted through videoconferencing due to experts being in different locations and was conducted in English, as this was the common language spoken amongst group members. The discussion employed a nominal group technique to generate ideas and have all voices heard. A single expert (TL) facilitated the discussion, starting by proposing the problem of how to document the process of data-driven health research in resource-limited contexts. All other experts then had a chance to contribute their ideas for building a process mapping tool, based on current evidence and their experience in global health research. They were able to propose new ideas or build on ideas that other experts shared. Specifically, experts were asked to focus on ideas for: key components of conducting health research studies, how these components may differ based on whether a health research study relies on primary versus secondary data, and what metrics are important to track throughout the health research study’s process. Subsequently, experts were asked to clarify and discuss their ideas further. Consensus was then reached through a public vote, defined as majority in favour (> 50%) of an idea. Components of the research tool were further refined based on this consensus, and a framework for the research tool was proposed. Figure 1 illustrates the process of this discussion.

Expert group discussions were not transcribed and further analysed, and accordingly, this process was exempt from ethics approval. Further, this consensus building exercise was not registered.

## Results

Informed by an expert group discussion, a practical approach was developed to help inform and drive the implementation of a uniform method to advance the quality and quantity of data-driven research in resource-limited settings through the documentation of common challenges, solutions, and tools. Accordingly, the foundations of the proposed approach lay in process mapping to document key steps undertaken, challenges encountered, solutions proposed, and tools used throughout individual health research studies, using a standardized framework. This add-on study approach was named “Pathfinder”.

At its core, a Pathfinder study is a research diary, where key processes of producing data-driven health research are mapped. Pathfinders track and process map what are referred to as “host studies”, which are actual health research studies that are either being planned, underway, or complete. One may conceptualize a host study as “what is being done”, and a Pathfinder study as “how it is being done”. Accordingly, we distinguish between two types of Pathfinders: retrospective and prospective. Namely, research teams can use Pathfinders to either retrospectively capture steps and processes in studies that have already culminated, or prospectively capture steps and processes as host studies are undertaken. The key difference is that prospective Pathfinders can be used for quality improvement as a host study is being undertaken, whereas retrospective Pathfinders intend to capture best practices from a host study that has already been completed.

**Fig. 2:**
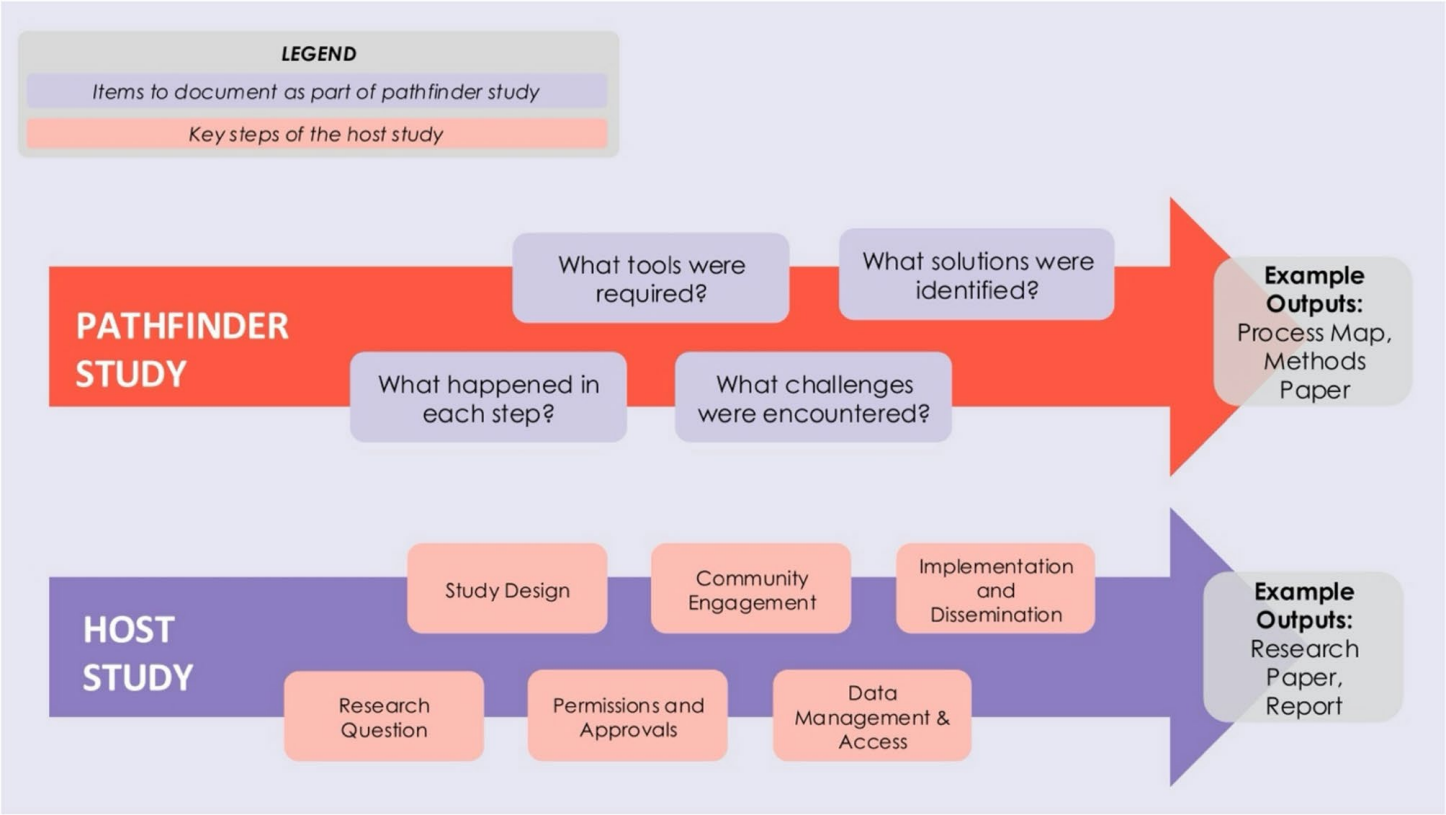
Using a Pathfinder study to track and map host studies

Figure 2 illustrates the difference between a host study and its associated Pathfinder study. Pathfinder studies may be carried out by members of the host study team, a different team, or a mixture.

Accordingly, a Pathfinder study, as an add-on to a host study, can be used to: [1] identify and record the component steps required for the host study to be completed successfully [2], track key metrics, such as time taken and resources required, for each of these component steps to generate a process map [3], support a prospective host study in identifying barriers and finding solutions that enable the achievement of their research objectives, and [4] capture and share the tools, methods, technology or governance processes used in solving challenges encountered in the host study.

Pathfinder teams are guided by a template protocol (S1 Appendix: Pathfinder Study Template Protocol) and may adapt this according to the specifics of their corresponding host study. This protocol may be used to obtain necessary approvals to conduct Pathfinder studies alongside host studies.

**Fig. 3:**
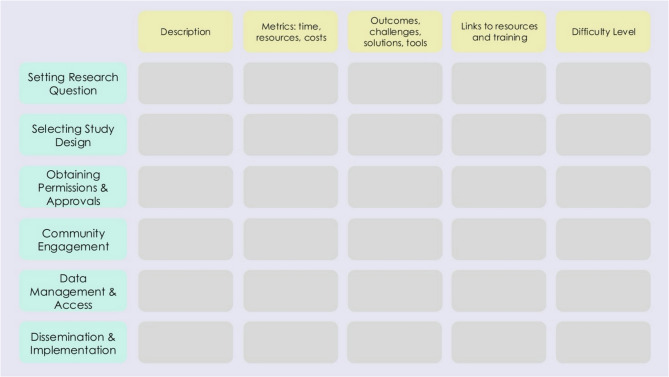
Basic Pathfinder study process mapping matrix

Figure 3 outlines a simple process mapping framework according to this protocol, which is elaborated on in S2 Appendix: Pathfinder Tracker. The rows in Fig. 2 depict key component steps, whereas the columns depict key elements tracked. This framework, called the Pathfinder study process mapping matrix, captures and tracks metrics on what activities took place to complete each step, what challenges arose, what solutions to those challenges were, what tools were used, and links to helpful resources and training that were required to achieve each step. In addition, Pathfinder teams are encouraged to comment on the difficulty level of achieving each component in the host study.

This framework can be adapted by Pathfinder teams and helps track the component steps that occur as a host study is designed, implemented, and reported. Component steps include elements such as setting the research question, selecting a study design, obtaining necessary approvals to undertake the host study, working with the community, designing and undertaking the analysis, generating high-quality evidence, and capturing how this evidence is to be taken up into policy and practice. Tracking component steps entails several things: [1] capturing key metrics such as time taken and resources required [2], recording how each step was undertaken [3], noting the tools and methods that enabled new evidence generation, and [4] capturing challenges and solutions.

**Fig. 4:**
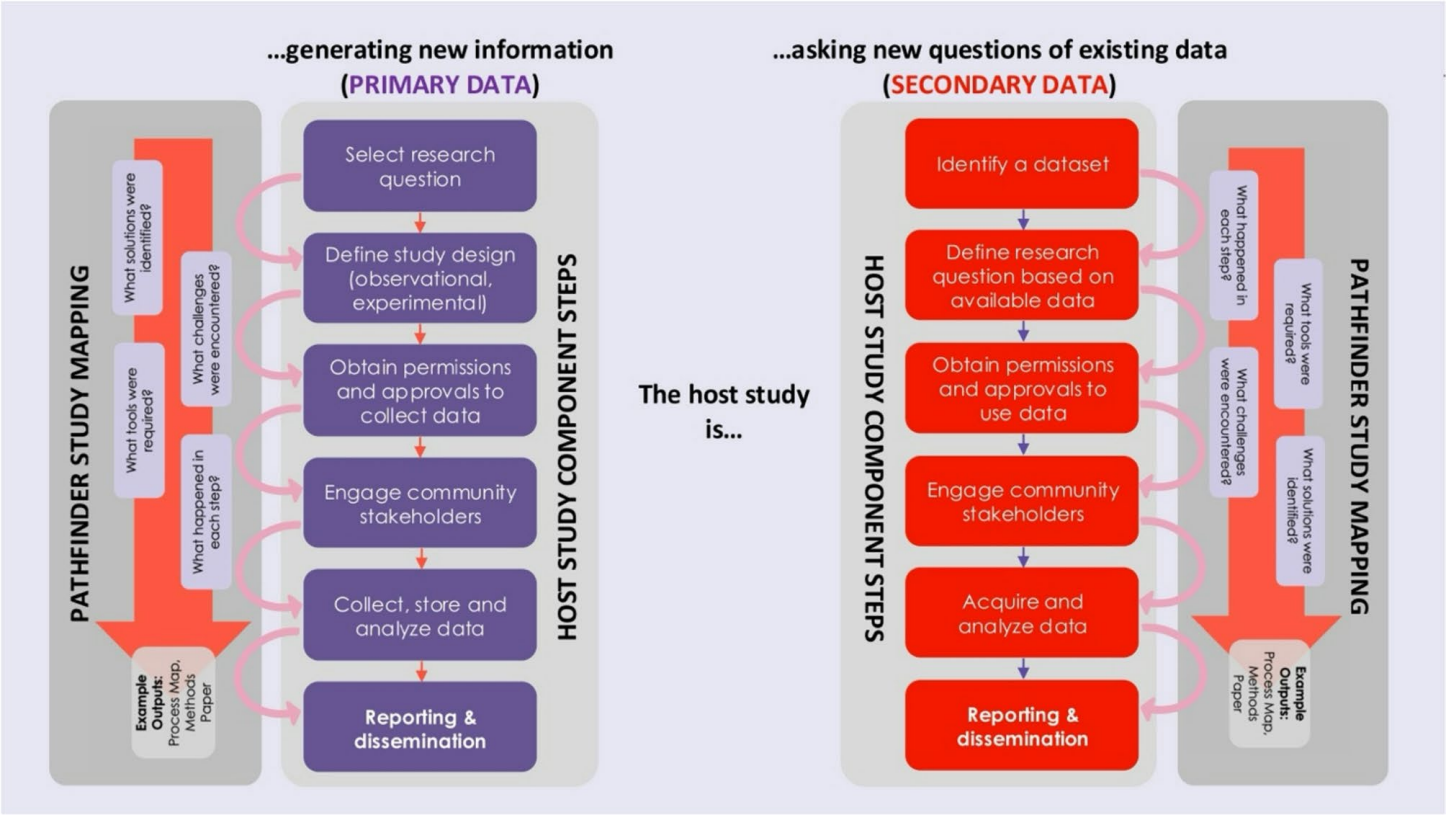
Schematic of two host studies: [1] primary research study and [2] a study that is asking new questions of existing data

Like we distinguished between two kinds of Pathfinder studies, it is important to distinguish two kinds of host studies. Firstly, host studies may ask new questions of existing data (secondary data), or secondly, they host studies may generate new information (primary data). We distinguish these two broad categories of host studies because their component steps look slightly different. Pathfinder studies that are mapping the process of asking new questions of existing data may focus more on data infrastructure and access, whereas those that are mapping the process of generating primary data may focus more on data collection. Figure 4 outlines key component steps in each type of host study, and how associated Pathfinder Studies may map these steps.

**Fig. 5:**
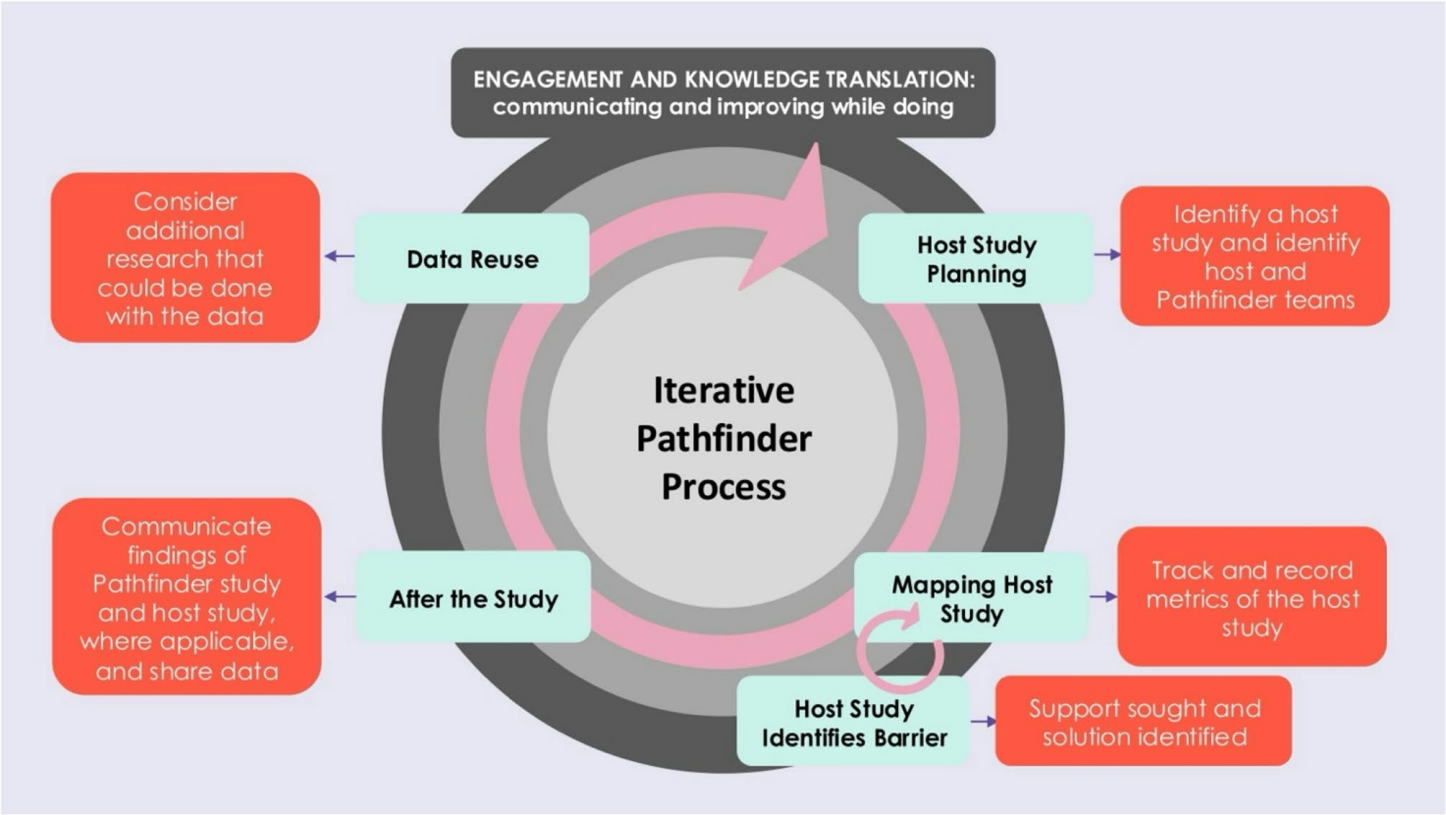
Iterative process of the Pathfinder method

As more Pathfinder studies are undertaken, through iterative learning-by-doing, and identifying when a roadblock is met, host study teams can be supported to identify a solution (Fig. 5). It is likely that another team will have met this roadblock before, and their solutions will have been tracked through the Pathfinder method. Consequently, the wider utility of aggerating findings from multiple Pathfinder studies are to: [1] build a database of process maps, categorized by host study design and topic area (e.g. epidemiology, social science, lab-based studies), and [2] build a database of host study steps, challenges, and solutions shared. Together, these objectives will help build global health research capacity by enabling high-quality data-driven health research.

## Discussion

Common areas of difficulty in conducting research have been documented before [2–4]; however, there has not been an equivalent effort to document common solutions, or indeed to share them between research teams working on different health challenges. Pathfinders offer a framework to both identify common challenges and share common solutions. This enables new evidence generation, raises research standards, and creates equity by sharing access to this know-how, particularly for researchers in low-resource settings [11–13]. Using the wider global health research community, through initiatives such as The Global Health Network, Good Research Practices, Epiverse, the Infectious Disease Data Observatory, MESH, and many more, may help identify specific solutions that research teams can use [10, 14–18].

Pathfinder process mapping fits well within a mixed-methods participatory research framework, and thus provides robust insight into the research team’s work related to their host study. It may be compared to other process mapping methods, such as flow charts, swim-lane diagrams, value stream mapping, multi-method process mapping, and time-driven activity-based costing, as well as process mapping tools, such as Microsoft Visio and Lucidchart [19–23]. Using these kinds of methods to map processes in healthcare more broadly has proven benefits in terms of understanding local systems and their nuances, informing future interventions, and exchanging knowledge [20]. The Pathfinder methodology uses similar principles to existing process mapping methodology, but tailors it to health research with a focus on data production, access, and use, and emphasizes the documentation of challenges related to conducting health research in resource-limited settings. It also underscores the importance of learning from others research teams through encouraging aggregation of Pathfinder studies, while other process mapping methods are often undertaken in isolation. Aggregated learnings from many Pathfinder studies will determine where there are common roadblocks in the process or operational delivery of data-driven health research and can help develop solutions for mitigating them.

While the Pathfinder methodology is adaptable to different study designs and settings, its strengths and pitfalls may be realized as it is implemented. As such, as the Pathfinder methodology is piloted in different settings and for different types of data-driven health research, we will continue to refine this method. We report that several global research institutes are piloting the Pathfinder methodology. These institutions are based in diverse locations across Latin America and the Caribbean (Brazil, Costa Rica, Honduras, Colombia), Asia (Bangladesh), and Africa (Nigeria), and are members of The Global Health Network, which is how they were selected for piloting. Host studies cover a variety of health research topics, such as maternal and child nutrition, birth outcomes, pathogen genomics, vaccine efficacy, and anti-microbial resistance. We intend to document the details and evaluation of the pilot Pathfinder studies in a separate paper.

It is also imperative to note the strengths and limitations of our own methodology in designing the Pathfinder tool. While we were able to convene expertise from diverse settings, we had representation from only six major health institutions around the globe. Accordingly, it is imperative that this tool is piloted in as many diverse contexts as possible. In addition, the expert group discussion consisted of a public vote, which could have biased results due to group-thinking. Finally, we did not employ more complex consensus-building methodology, like the Delphi technique, and did not employ multiple rounds of consensus building, which could help further refine ideas. Nonetheless, we believe that Pathfinders have strong potential to increase both quality and quantity of data-driven health research in resource-limited settings.

Indeed, health research methodology is rapidly evolving, enabling more complex surveillance, observational studies, and clinical trials. Applications of these new approaches must not remain in predominantly wealthy settings with access to resources, but also be applied across low-resource settings and in communities and disease threats underserved by research. We hope that Pathfinder studies will serve to determine how these new methods can be undertaken in varied contexts, uncover common barriers, and find common solutions, to help drive health research where it is needed most.

## Conclusion

Global health research experts convened to develop and propose a novel methodology, called “Pathfinder”, as a means of tracking and mapping the processes of undertaking data-driven health research. By documenting the key components, challenges, solutions, and tools at each stage of the health research process, Pathfinder studies can capture best practices, thereby removing barriers to health research, and addressing global inequality in this domain. As this methodology is piloted in several institutions around the world, we underscore its potential in advancing both quality and quantity of data-driven health research led by teams in resource-limited settings.

## Electronic supplementary material

Supplementary Material 1

## Data Availability

No datasets were generated or analysed during the current study.

## Abbreviation

LAC: Latin America and the Caribbean
