# Supplementary material for: Pathfinder studies: a novel tool for process mapping data-driven health research to build global research capacity
Source: BMC Med Res Methodol. 2025 Aug 7;25:190. doi: 10.1186/s12874-025-02638-7 (PMC12329893; doi:10.1186/s12874-025-02638-7)
Supplement: Supplementary file 1 — Supplementary Material 1 [file 12874_2025_2638_MOESM1_ESM.docx]

**S1 Appendix: Pathfinder study Template Protocol**

1. **TITLE**

A processing mapping study to determine the challenges and solutions in **[generating new evidence from existing data / an observational study / a surveillance study / a clinical trial / a social science study / other – describe study type]** to that aims to **[primary outcome of the host study]**

1. **BACKGROUND AND RATIONALE**

There is missing evidence to understand how to manage, treat and prevent **[disease or health condition of interest].** There is **[an existing dataset / a primary health research study]** that **[could generate / has generated]** new evidence that **[describe aim of the host study].**

Generating new evidence from health research studies and existing datasets can be challenging, and difficulties arise in teams undertaking the design, operational delivery, analysis, and dissemination of health research. It is particularly difficult to have research findings turned into recommendations that can be taken up into policy and practice. The Pathfinder methodology is an add-on study that can identify component steps of health research, and track key challenges and associated solutions. The aims are learning from this process, documenting and capturing the steps systematically, identifying problems or system hurdles, and finally, designing relevant solutions to resolve these hurdles. Therefore, this Pathfinder study will be an ‘add on’ to the host study, whether this is to assist in undertaking new analysis of existing datasets, or to support studies that are capturing primary health research data.

Because the Pathfinder methodology fits within an action research framework, it employs an iterative learning-by-doing, pragmatic approach. This iterative approach ensures the success of the host study in generating safe, accurate and ethical new evidence, by tackling challenges and refining processes as they occur. Identified processes, challenges, lessons and solutions will be shared widely so that others can adopt them to generate new evidence from existing their studies and existing datasets in other disease areas and health challenges. This will be done through the communities of practice built across The Global Health Network and partners.

1. **AIMS**

Overall, this ‘add-on’ process-mapping study aims to record and track the steps of the ‘host’ study, **[insert title],** to determine the challenges and successes encountered in the generation of evidence. Specific aims are to (1) generate a process map of the host study to document all the required steps and their associated metrics; (2) to determine the tools, methods, approaches, and systems that were applied to each step, and (3) to describe each challenge encountered and document how these were solved.

1. **METHODOLOGY**
   1. **Selection of Host Study**

Any plan for a health-related study that aims to generate new evidence, or any plan to extract new evidence from an existing health-related dataset could serve as a host for an add-on study such as this. The overall aim is to track the process of the host study, which may be of several types of designs, such as surveillance, observational, interventional, social science and behavioural studies. For studies involving new analysis of existing data; host studies could explore vast datasets held across different geographies or organisations. On the other hand, datasets could be small and simple, held by a healthcare team wishing to gain new insights from that data to improve clinical practice.

Pathfinder studies are embedded in an action research framework, and therefore, the host study could benefit as processes can be refined as the Pathfinder study documents and learns from challenges. Or, the host study team might be very experienced and can therefore share the benefit of this add-on study by recording and sharing best practices with others. These add-on studies can be undertaken by a small subset of the host study team, or another small group where the host study team is willing to embed them in their operations to track and document the host study process. In this section of the template protocol, the Pathfinder study team should describe the host study and why there is benefit in adding on a process-mapping Pathfinder study. **[Add host study description and rationale for Pathfinder study (why/how the host study was chosen, who is undertaking the Pathfinder study, and why the Pathfinder study is needed)].**

- 1. **Organisation of Teams and Operational Delivery**

These Pathfinder studies can also serve as a research capacity and career development opportunity. It can be that this Pathfinder study is led and coordinated by the host study team itself, and can be a strong development opportunity for someone within the team to take on this responsibility and gain experience within methodology research. Alternatively, the Pathfinder study team could be set up elsewhere by collaborators or have co-investigators where there is existing expertise in data science and methodology research. Once again, due to the action research nature of the Pathfinder methodology, the process of running these add-on studies should be highly beneficial for the host study team in that this will identify and solve their challenges iteratively. Therefore, the Pathfinder study should represent the host study and address any missing expertise. This section of the template protocol should set out the Pathfinder study team and their roles and responsibilities. **[Add details related to host study team and Pathfinder study team].**

- 1. **Process Mapping a Study Using the Pathfinder Method**

A matrix will be used as a data capture form that can be adapted to each study to firstly capture all the steps required of the host study being process mapped, the quantitative metrics of these steps, the associated qualitative information about how each encountered challenge was solved, and finally the capture the tools, training, method or technology used as a document or weblink. See S2 Appendix: Pathfinder Tracker for details. Importantly, the tracker may be varied to reflect the specifics of each host study; here are the component steps of two examples of Pathfinder studies that could be set up within the matrix:

4.3.1 Add-on to a primary research study:

- Setting the primary objectives (setting the questions).
- Deciding on what is to be measured to answer the question, how and when.
- Study management, governance and compliance.
- Selecting the population and setting.
- Community engagement and involvement.
- Designing the study operations.
- Assuring quality, safety and ethical standards.
- Data management, analysis, sharing.
- Taking the findings into recommendation for policy and practice.

4.3.2 Add-on to a study that is asking new questions of existing data:

- Identifying a dataset – or several datasets that can be analysis.
- Defining the research question.
- Defining and characterising the type of data to answer the ‘add-on’ research questions.
- Understanding who is the custodian of the data and if there are other stakeholders.
- Working with the community to refine the research question and determine the value of the evidence.
- Reviewing and ensuring that the governance, ethical and other permissions are in place.
- Wider resource mapping including team skills, capacity and tools required to carry out research project.
- Determining what is needed to prepare the data for analysis such as extraction, quality control, standardisation, and curation.
- Agreeing data management plan including access to data, storage requirements, and future data access and output sharing following FAIR principles.
- Agreeing the analysis plan and undertaking the analysis.
- Setting and monitoring metrics for data cycle add-on methodology.
- Taking the findings into practice and policy.

In this section, the Pathfinder study team will include their tracking matrix as a table. Pathfinder study teams may wish to highlight common themes seen in their tracker. **[Pathfinder study team to add Pathfinder tracker and key findings here].**

- 1. **Qualitative Evidence to Understand the Steps, Processes, and Challenges**

This section will capture the qualitative elements that can then explain and further understanding of quantitative metrics. Here, the Pathfinder study team will decide whether to use interviews, questionnaires or focus groups to gain insight into the decisions, challenges and solutioning around each step, process, and challenge. This could include attending the host study’s team meetings and completing a standard form for each, asking the host study team to complete a regular questionnaire to capture their issues and challenges, or undertake interviews or focus group discussions with the host study team. This is the mechanism for determining the ‘who, where, what’ explanation for each step, success and challenge identified in the previous section. It is recommended to use guiding questions to assist focus groups and individual interviews. These questions can be elaborated according to the reality of the host study, considering the time available to attend the data collection sessions and the profile of the attendees. Semi-structured interviews are advisable, since the participants can bring new and interesting details on the host study that were not anticipated by the Pathfinder team, but that can enrich the final impact of the study. It is also recommended to invite representatives from all hierarchical levels of the host study, to make sure different perspectives are taken into account and all voices are heard. **[Pathfinder study team to add details related to obtaining information listed in the Pathfinder Tracker].**

- 1. **Documentation of Tools, Processes, Methods, Training**

This is where the Pathfinder study team can record the specific tools, methods, training or resources found, used, developed, or adapted for each host study step. These can then be tagged and added to the tracker and be shared with others for wider impact, as perhaps a guidance document, training course, template or weblink to technology, further training, etc. **[Pathfinder study team to add details related to tools, processes, methods, and training, and to include references to any external training used]**.

- 1. **Solving Challenges and Connecting Excellence**

The Pathfinder study’s mixed methods action research nature means that it aims to iteratively identify problems and solve them; thereby learning-by-doing. The Pathfinder process can work to find solutions for the challenges encountered by the host study. This could be done by identifying training, templates, data science software or infrastructure, or documents and guidance for data sharing agreements. Such tools solutions could be found across the communities of practice for data-driven research, such as The Global Health Network Data Science Hub, Applied Epi, Health Data Research Global, and the European & Developing Countries Clinical Trials Partnership, for example.

Where no solution can be found, the Pathfinder study team can work to find experts by asking across these open communities. Ultimately, if there is no solution, this is also an important finding and further supports the need for this process mapping to document key gaps. **[Pathfinder study team to describe how key challenges were solved].**

1. **DATA ANALYSIS**
   1. **Generation of Study-Specific Process Map Diagram with Metrics**

There will be simple time-related metrics associated with each host study step that can allow for the creation of a study-specific process map. This will be visualised to show the relationship, sequence, and order of each host study step. Additional quantitative metrics such as resources required, and step difficulty level may be added to this process map. **[Pathfinder study team to include a process map figure here].**

- 1. **Qualitative Analysis**

Here, the Pathfinder study team may wish to perform a thematic analysis of questionnaires, forms, interviews, and focus groups that helped them identify barriers and solutions in each host study step. **[Pathfinder study team to include findings from qualitative analysis here].**

1. **INTERPRETATION AND USE OF FINDINGS**

The Pathfinder study’s action research nature lends itself to interactive support between the Pathfinder study team and host study team in real-time. Any maps and tools generated through the Pathfinder process can be shared with the host study team to improve research activities. Ultimately, these Pathfinder study outputs would feed into a larger analysis of many Pathfinder studies to generate guidance for research teams around the world. **[Pathfinder study team to describe which outputs were created and how they were used to benefit the host study].**

1. **ETHICAL CONSIDERATIONS**

The Pathfinder study is a type of process tracking methodology that can be described as an audit. It will map the steps of the host study, which should already have primary ethical clearance, if applicable. The process mapping will only measure, report activities and offer insights into host study conduct. No patient, public or participant information or data will be accessed, seen, or used within this Pathfinder study. The datasets generated will be metrics related to the host study’s processes and qualitative information from the study staff about their experience regarding these study steps. Ethics committees and host institutions will be notified of this audit-type study for their information. There is no risk to the participants in the original study as they, and their data, are not involved in this process.

1. **COMMUNICATION AND DISSEMINATION OF THE FINDINGS**

The following steps should be taken by the Pathfinder study team to disseminate findings:

1. The Pathfinder Tracker and Protocol will be developed and shared through The Global Health Network’s Data Science and regional knowledge hubs.
2. Real-time iterative sharing of methods and host study process improvement steps will be enacted where practical and possible. Ultimately, generalised process maps from the different Pathfinder studies will be communicated in real-time to the host study teams for iterative improvement and excellence sharing.
3. Outputs will be shared on The Global Health Network’s platform. This is an open community of practice for sharing research methods, skills and processes. Outputs will be shared in numerous places on the platform, such as knowledge hubs, the data science hub, and within The Global Health Network’s training centre.
4. Outputs may be shared through other networks and the host study’s communication platforms as well.

**S2 Appendix: Pathfinder Tracker**

| **Component Steps** *Can adapt these or add more based on type of host study* | **Description** | **Metrics** | | | | **Qualitative responses^2^** | **Links to resources, tools, training** |
| --- | --- | --- | --- | --- | --- | --- | --- |
|  |  | **Started** | **Completed** | **Number of Weeks** | **Number of Resources^1^** |  |  |
| Identify research question |  |  |  |  |  |  |  |
| Identifying relevant datasets |  |  |  |  |  |  |  |
| Developing research protocol |  |  |  |  |  |  |  |
| Completing funding and award letters |  |  |  |  |  |  |  |
| Team, skills and tools planning |  |  |  |  |  |  |  |
| Accreditation of researchers |  |  |  |  |  |  |  |
| Undertaking ethical and impact approval |  |  |  |  |  |  |  |
| Developing data management plan |  |  |  |  |  |  |  |
| Data governance considerations |  |  |  |  |  |  |  |
| Data capture and collection |  |  |  |  |  |  |  |
| Data characterisation and quality assessment |  |  |  |  |  |  |  |
| Data collation, curation, cleaning and preparation |  |  |  |  |  |  |  |
| Making datasets more FAIR (Findable, Accessible, Interoperable, Reusable) |  |  |  |  |  |  |  |
| Developing data analysis plan |  |  |  |  |  |  |  |
| Undertaking exploratory data analysis |  |  |  |  |  |  |  |
| Choosing appropriate statistical models |  |  |  |  |  |  |  |
| Undertaking analysis and validating results |  |  |  |  |  |  |  |
| Developing stakeholder engagement plan |  |  |  |  |  |  |  |
| Engagement activity involved in the project |  |  |  |  |  |  |  |
| Developing publication and dissemination plan |  |  |  |  |  |  |  |
| Preparation of output |  |  |  |  |  |  |  |
| Publishing research output (e.g. code on GitHub, tools online, papers published) |  |  |  |  |  |  |  |
| Preparation of publication |  |  |  |  |  |  |  |
| Submission and review research publication |  |  |  |  |  |  |  |
| Monitoring and evaluation of research impact |  |  |  |  |  |  |  |
| Dissemination activity involved in the project |  |  |  |  |  |  |  |

*Different shading represents six broad categories: 1) setting your research question, 2) study design and planning, 3) approvals and permissions, 4) data management, 5) stakeholder and community engagement, 6) dissemination and implementation.*

*^1^ Some things you may want to note here are: What were the costs? Who was involved? What was the difficulty level of this step?*

*^2^ Some things you may want to note here are: What deliverables/step outputs were achieved? What skills were needed? What tools were needed? What were the challenges or barriers you faced? How were these challenges resolved? What lessons have you learned from these experiences? What would you do differently?*
